# Supplementary material for: A versatile oblique plane microscope for large-scale and high-resolution imaging of subcellular dynamics
Source: eLife. 2020 Nov 12;9:e57681. doi: 10.7554/eLife.57681 (PMC7707824; doi:10.7554/eLife.57681)
Supplement: Supplementary file 2. [file elife-57681-supp2.docx]

| **Probe names** | **Gene specific sequence - detection probe specific sequence** |
| --- | --- |
| hsHR2X-ACE2-1719 | aatgctagggtccagggttc TTATACGTCGAGTTGAACGTCGTAACA |
| hsHL2X-ACE2-1719 | TAGCGCTAACAACTTACGTCGTTATG tgattttccaagcctcagca |
| hsHR2X-ACE2-58 | agcagttacagcaacaaggc TTATACGTCGAGTTGAACGTCGTAACA |
| hsHL2X-ACE2-58 | TAGCGCTAACAACTTACGTCGTTATG tgagaaggagccaggaagag |
| hsHR2X-ACE2-1457 | ctcgcttcatctcccaccac TTATACGTCGAGTTGAACGTCGTAACA |
| hsHL2X-ACE2-1457 | TAGCGCTAACAACTTACGTCGTTATG tttttcatccactggtcttt |
| hsHR2X-ACE2-2872 | atgcatgccattctcaatcc TTATACGTCGAGTTGAACGTCGTAACA |
| hsHL2X-ACE2-2872 | TAGCGCTAACAACTTACGTCGTTATGttgcagctacaccagttccc |
| hsHR2X-ACE2-1038 | actgctttctgaacatttcc TTATACGTCGAGTTGAACGTCGTAACA |
| hsHL2X-ACE2-1038 | TAGCGCTAACAACTTACGTCGTTATGtgggtccgttagcatggaat |

**Supplementary File 2.** Encoding probe sequences for proximity ligation RNA fluorescence *in situ* hybridization.
