# Supplementary material for: A versatile oblique plane microscope for large-scale and high-resolution imaging of subcellular dynamics
Source: eLife. 2020 Nov 12;9:e57681. doi: 10.7554/eLife.57681 (PMC7707824; doi:10.7554/eLife.57681)
Supplement: Supplementary file 3. [file elife-57681-supp3.docx]

| **Cell Line** | **Source** | **Authentication** | **Testing** |
| --- | --- | --- | --- |
| MV3 | Friedl Lab | N/A | Mycoplasma negative |
| U2OS | ATCC (HTB-96) | N/A | Mycoplasma negative |
| RPE hTERT | ATCC (CRL-4000) | N/A | Mycoplasma negative |
| ARPE | ATCC (CRL-2302) | N/A | Mycoplasma negative |
| NK-92 | ATCC (CRL-2407) | N/A | Mycoplasma negative |
| K562 | ATCC (CCL-243) | N/A | Mycoplasma negative |
| 1205Lu | Wistar | STR Fingerprint | Mycoplasma negative |
| MEFs | ATCC (SCRC-1040) | N/A | Mycoplasma negative |

**Supplementary File 3.** Source, authentication method, and routine testing performed on cell lines.
